# Novel variants related to extreme elevation of serum IgE in Vietnamese patients with primary immunodeficiency: Case report

**DOI:** 10.1097/MD.0000000000045565

**Published:** 2025-11-07

**Authors:** Le Thi Minh Huong, Nguyen Thi Kim Lien, Nguyen Thi Van Anh, Tran Minh Dien, Nguyen Thi Phuong Mai, Ngo Diem Ngoc, Nguyen Minh Thu, Nguyen Thi Thanh Ngan, Nguyen Thanh Hien, Nguyen Van Tung, Nguyen Huy Hoang

**Affiliations:** a Vietnam National Hospital of Pediatrics, Ministry of Health, Hanoi, Vietnam; b Institute of Biology, Vietnam Academy of Science and Technology, Hanoi, Vietnam; c Graduate University of Science and Technology, Vietnam Academy of Science and Technology, Hanoi, Vietnam; d Phenikaa University Hospital, Ministry of Health, Vietnam.

**Keywords:** hyper-IgE, novel variants, primary immunodeficiency (PID), Vietnamese patients, whole-exome sequencing (WES)

## Abstract

**Rationale:**

Extreme elevation of serum immunoglobulin E (IgE) concentration is a key marker for detecting immune disorders, including humoral and cellular defects in primary immunodeficiency (PID). IgE antibodies are present in low concentrations in the body and are produced in large amounts when exposed to infections or toxins. However, IgE is also the cause of allergic symptoms and life-threatening anaphylaxis reactions. Early diagnosis of PID associated with elevated IgE may lead to effective or life-saving therapeutic interventions. Therefore, genomic testing-based diagnosis is becoming a widely used diagnostic tool to determine the cause of disease.

**Patient concerns:**

Three Vietnamese patients with increased IgE expression were collected for genetic analysis at The Allergy, Immunology, and Rheumatology Department, Vietnam National Hospital Pediatrics.

**Diagnoses:**

Primary immunodeficiency associated with elevated IgE.

**Interventions:**

We performed whole-exome sequencing (WES) to detect novel associated variants and confirmed these by Sanger sequencing. The effects of the variants were predicted using in silico prediction tools.

**Outcomes:**

Three novel pathogenic variants including c.2204A > T, p.Asp735Val in the *PTPRC* gene, c.586T > A, p.Phe196Ile in the *UNC119* gene, and c.481C > T, p.Arg161Cys in the *IL21R* gene were found to be associated with increased IgE.

**Lessons:**

We report novel variants associated with genetic defects that increase IgE found in PID patients. These results emphasize the need for accurate diagnosis and appropriate intervention to improve outcomes and quality of care for individuals with high IgE levels and related immune disorders.

## 1. Introduction

Primary immunodeficiency (PID) is a diverse group of rare genetic disorders that consist of monogenic immunoregulatory disorders.^[1]^ PID involves quantitative and/or functional disorders in the immune system that can lead to a higher risk of infection, immune dysfunction, autoimmune disorders, and inflammation.^[2]^ Elevated serum IgE levels have been associated with an increasing number of PID syndromes, including humoral, cellular, severe combined immunodeficiency (SCID), and other defects in T-cell development.^[3,4]^ In addition, other PIDs associated with extreme elevation of IgE include hyper-IgE syndrome, Comèl Netherton syndrome (NS), and allergy disorders have been reported.^[5]^

Many studies have been conducted to identify the causes of hyper-IgE; however, understanding remains challenging due to the phenotypic overlap between PID forms and allergies; with characteristics of hyper-IgE may also lead to delays in diagnosis.^[6]^ Several single-gene disorders associated with IgE overproduction have been discovered over the past 2 decades, each requiring different clinical considerations and treatment approaches.^[7]^ Previous studies have implicated genes involved in elevated serum IgE levels in many syndromes/diseases, include barrier defects (*CDSN, DSG1, FLG, SPINK5,* and *TRPV3*), cytokine signaling defects (*ERBIN, IL6R, IL6ST, IRAK4/MyD88, STAT3, STAT5b, TYK2,* and *ZNF341*), defective glycosylation (*PGM3*), impaired T cell receptor (TCR) signaling and cytoskeletal remodeling (*ARPC1B, CARD11, DOCK8, MALT1*, and *WAS*), primary immunodeficiencies (*DCLRE1C, PGM3, PLCG2, SPINK5, STAT3, TGFBR1*, and *TGFBR2*), restricted T‐cell repertoire (*ADA, CHD7, IL2R, IL7R, RAG1, RAG2*, and *ZAP70*), SCID (*ADA*), tolerance failure (*ALPS* and *FOXP3*).^[4,8]^

Despite the increasing number of reported genes associated with hyper-IgE, the mechanism of hyper-IgE remains poorly understood. A study by Alyasin et al^[9]^ of 18 patients with hyper-IgE syndrome found that 10 carried pathogenic variants in the *DOCK8* gene, 4 carried variants in the *STAT3* gene, and 4 had unknown variants. This highlights the implications for our understanding of the pathogenesis and clinical management of patients with these complex and challenging forms of PID.^[10]^ Early and accurate diagnosis of the variants associated with each form of PID can lead to effective medical interventions and treatments for patients.^[4]^ Over the past decade, molecular diagnostics, especially next-generation sequencing, have contributed to the accurate diagnosis and elucidation of novel genetic defects associated with elevated IgE levels.^[11]^

In this study, we performed whole-exome sequencing (WES) to identify variants that cause IgE over-synthesis in Vietnamese patients with PID. Information on the variants will help to understand the etiology of the disease and contribute to a general understanding of the pathogenesis in patients.

## 2. Case presentation

### 2.1. Ethics statement

The study was conducted by the Declaration of Helsinki and was approved by the Ethics Committee of the Institute for Genome Research (Approval No. 02-2024/NCHG-HDDD). Informed consent was obtained from the patient’s parents to publish images or data included in the article.

### 2.2. The proband

Three patients from unrelated families have been diagnosed with PID at the Allergy, Immunology, and Rheumatology Department, Vietnam National Hospital Pediatrics. Clinical information of patients is detailed in Table 1.

**Table 1:** Clinical information of patients.

Patient	PID1	PID2	PID3
Sex	Male	Male	Female
Age of onset	5 yr	1.5 mo	1 mo
Pneumonia	x	x	
Otitis media		x	
Diarrhea		x	
Sepsis		x	x
Dermatitis		x	x
Lymph nodes	x		

PID = primary immunodeficiency.

Patient 1, a boy, was admitted with pneumonia and generalized lymphadenopathy at the age of 5 years. The IgE concentration measured in the patient was 9891 IU/mL, much higher than that of normal children of the same age (normal range is 2–307 IU/mL). Patient 2 was a 1.5-month-old boy who was admitted to the hospital with pneumonia, sepsis, dermatitis, otitis media, and persistent diarrhea. The measured IgE level was 898 IU/mL, which was higher than normal (normal level is < 15 IU/mL). Patient 3 was a 1-month-old girl who was admitted to the hospital with severe sepsis and dermatitis. Her IgE level was 1481 IU/mL, and she went into septic shock and died.

## 3. Materials and methods

### 3.1. Genetic analysis

Genomic DNA was extracted using a Qiagen DNA blood mini kit (QIAGEN, Hilden, German) and concentrated using a Thermo Scientific NanoDrop spectrophotometer (Waltham). WES was performed using the Agilent SureSelect Target Enrichment kit (Agilent) for library preparation and the SureSelect V7-Post kit (Illumina, CA, USA) for sequencing in the Illumina platform (Illumina, CA, USA). The data were analyzed using bioinformatic software to determine, annotate, and predict the effects of variants. Variants were screened for likely pathogenic and/or novel, rare variants (with low MAF < 0.001) in disease-associated genes. Variants were validated by Sanger sequencing on the patient sample and family members. Sanger sequencing was conducted on an ABI PRISM 3500 Genetic Analyzer machine (Thermo Fisher Scientific Inc., USA).

### 3.2. In silico analysis

The influence of the novel variants was evaluated with the *in silico* analysis tools: CADD (https://cadd.gs.washington.edu/snv), Fathmm (https://fathmm.biocompute.org.uk/fathmmMKL.htm), Mutation Taster (https://www.genecascade.org/MutationTaster2021/), PANTHER (http://www.pantherdb.org/tools/), PolyPhen 2 (http://genetics.bwh.harvard.edu/pph2/), PON-P2 (https://bio.tools/pon-p2), PROVEAN (http://provean.jcvi.org/seq_submit.php), SIFT (https://sift.bii.a-star.edu.sg/), and SNP&GO (https://snps-and-go.biocomp.unibo.it/snps-and-go/). The 3-dimensional structure of the wild type and mutant variants were constructed using Swiss-PDB Viewer v4.1 with PDB: P08575 (for PTPRC protein) and Q9HBE5 (for IL21R protein).

## 4. Results

This study investigated WES in 3 PID patients diagnosed with unexplained serum IgE levels (Table 2). Patient 1 had clinical symptoms of pneumonia and lymph node abscess, and laboratory results showed increased serum IgA and IgM levels along with eosinophil and lymphocyte counts. Patient 2 had symptoms of pneumonia, dermatitis, diarrhea, and sepsis with a decreased IgG concentration and T lymphocyte count. Patient 3 had severe dermatitis and sepsis, and the patient developed septic shock, leading to death. Immunological tests showed that the indices were all within the normal range, while IgG and CD8 + increased. This result showed that the patients in the study did not have typical symptoms of syndromes leading to increased IgE, such as hyper-IgE syndrome (HIES), Netherton syndrome (NS) or Comel-NS, Omenn syndrome (OS), immune dysregulation polyendocrinopathy enteropathy X-linked syndrome (IPEX), and Wiskott-Aldrich syndrome (WAS).

**Table 2:** Paraclinical findings in patients.

Patient	PID1	PID2	PID3
Serum immunoglobulin concentrations			
IgA (g/L)	0.98	1.04	0.22
IgE (UI/L)	**9891.3**	**898**	**1481**
IgM (g/L)	1.74	1.18	0.37
IgG (g/L)	13.71	7.30	**1.08**
Flow cytometry indicators			
CD3 (abs, %)	**4877** (60.8)	**1719** (21.4)	5141 (73.0)
CD4 (abs, %)	**2663** (33.0)	**941** (11.7)	3058 (43.0)
CD8 (abs, %)	**1883** (23.0)	**619** (7.6)	1855 (26.0)
CD19 (abs, %)	**1917** (23.0)	1315 (15.8)	1139 (16.0)
CD56 (abs, %)	**1153** (14.3)	440 (5.5)	696 (9.9)

Reference ranges for serum immunoglobulin according to Tosato et al.^[12]^ Reference ranges for lymphocytes subsets according to Bayram et al.^[13]^ Normal level in 1–3 mo healthy children: IgA = 0.067–0.246 g/L; IgM = 0.152–0.685 g/L; IgG = 2.170–9.810 g/L; IgE < 60 IU/mL. CD3 = 3180–5401 n × 10^6^/L; CD4 = 2330–3617 n × 10^6^/L; CD8 = 712–1361 n × 10^6^/L; CD19 = 315–1383 n × 10^6^/L; CD56 = 201–870 n × 10^6^/L. Normal level in 3–5 yr healthy children: IgA = 0.357–1.920 g/L; IgM = 0.587–1.980 g/L; IgG = 4.570–11.200 g/L; IgE < 15 IU/mL. CD3 = 1578–3707 n × 10^6^/L; CD4 = 870–2144 n × 10^6^/L; CD8 = 472–1107 n × 10^6^/L; CD19 = 434–1274 n × 10^6^/L; CD56 = 155–565 n × 10^6^/L. The bold values indicate values that exceed the normal range.

IgA = immunoglobulin A, IgE = immunoglobulin E, IgG = immunoglobulin G, IgM = immunoglobulin M, PID = primary immunodeficiency.

We performed WES on 3 PID patients with elevated IgE levels to detect pathogenic variants in relevant genes. We detected 3 novel variants in the *PTPRC, UNC119*, and *IL21R* genes, which were potentially involved in the pathogenesis, for evaluation by prediction software and Sanger sequencing. Evaluation showed that all identified variants have high pathogenic potential (Table 3). Variant c.2204A > T, p.Asp735Val, in the *PTPRC* gene, was evaluated as pathogenic by all software. Variant c.586T > A, p.Phe196Ile in the *UNC119* gene was also evaluated as pathogenic by all software (CADD, Fathmm, Mutation Taster, PolyPhen-2, and SNP&GO) except SIFT software with tolerance. Variant c.481C > T, p.Arg161Cys in the *IL21R* gene are considered pathogenic by PolyPhen-2, SIFT, and SNP&GO software, but as neutral by CADD and benign by Fathmm and Mutation Taster software.

**Table 3:** Variants in PID patients with hyper-IgE.

Patient	PID1	PID2	PID3
Gene	*PTPRC* OMIM151460 1q31.3-q32.1	*UNC119* OMIM604011 17q11.2	*IL21R* OMIM605383 15p12.1
cDNA	c.2204A > T	c.586T > A	c.481C > T
Protein	p.Asp735Val	p.Phe196Ile	p.Arg161Cys
Zygosity	het	het	het
dbSNP/MAF	novel	novel	novel 0.00239617
In silico prediction tools			
CADD	Damaging (28.3)	Damaging (29.7)	Neutral (16.20)
Fathmm	Damaging (0.967)	Damaging (0.962)	Benign (0.041)
Mutation Taster	Deleterious	Deleterious	Benign
PANTHER	Probably damaging (0.85)	–	–
PON-P2	Pathogenic (0.943)	–	–
Poly Phen-2	Probably damaging (1.000)	Probably damaging (0.989)	Probably damaging (0.990)
PROVEAN	Deleterious (−8.394)	–	–
SIFT	Disease (0.000)	Tolerant (0.103)	Disease (0.034)
SNP&GO	–	Disease (RI: 6)	Disease (RI: 2)

IgE = immunoglobulin E, MAF = minor allele frequency, PID = primary immunodeficiency.

## 5. Discussion

In this study, 3 patients diagnosed with PID in unrelated Vietnamese families were recruited for genetic analysis. All patients had elevated serum IgE levels but did not have typical clinical and laboratory features of known syndromes leading to increased IgE (Table 2). According to previous reports, high serum IgE levels and eosinophilia are present in most patients with HIES. In contrast, IgG levels are normal or elevated, and IgM levels are often decreased.^[14]^ Stuvel et al^[15]^ reported on 14 patients with NS syndrome who showed normal serum IgA, IgM, and IgG levels and lymphocyte counts. This result was also reported in patients with OS syndrome.^[16,17]^ Patients with IPEX syndrome have normal IgA, IgG, and IgM levels^[18,19]^ but increased CD3 + and CD4 + levels.^[20,21]^ In WAS patients, IgA and IgE levels are reported to be increased, while IgG and IgM levels are decreased.^[22]^ However, in a case report of a 10-month-old boy with WAS, his immunologic profile showed elevated levels of IgE and IgA but normal IgG and IgM levels.^[23]^

Elevated serum IgE levels were considered a common marker of severe immune disorders,^[24]^ especially inborn errors of immunity^[25]^ and associated with life-threatening immune overreactions.^[26]^ Immune disorders that result in increased serum IgE concentrations have been identified as being caused by single genes, and each disorder requires different clinical care and treatment approaches. This highlights the need for accurate diagnosis to enable appropriate intervention, improving treatment outcomes for patients with high IgE levels due to immune-related disorders.^[7,25]^ However, the underlying cause of increased serum IgE levels remains unclear, and many immune disorders are often misdiagnosed as a common allergies. Distinguishing between individuals with elevated serum IgE levels due to common allergies and inborn errors of immunity is challenging because of the influence of different genetic and environmental factors.^[27,28]^

**Figure 1.**
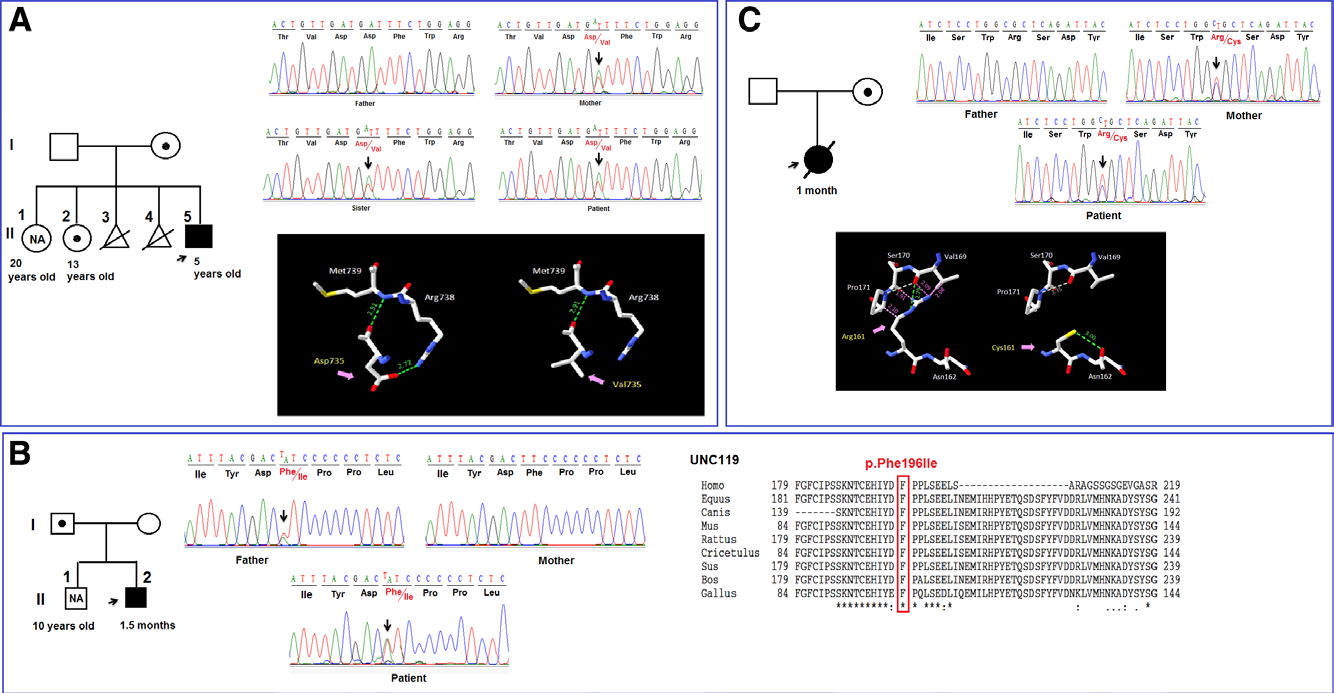
Variant analysis in patients in the study. (A) Pedigree of the P1 patient’s family; Sanger sequencing results at position c.2204A > T in the *PTPRC* gene; 3-dimensional structure of the PTPRC protein predicted by Swiss-PdbViewer. (B) Pedigree of the P2 patient’s family; Sanger sequencing results at position c.586T > A in the *UNC119* gene; alignment of amino acid sequences of UNC119 from different species. (C) Pedigree of the P3 patient’s family; Sanger sequencing results at position c.481C > T in the *IL21R* gene; 3-dimensional structure of the IL21R protein predicted by Swiss-PdbViewer.

In this study, we identified 3 novel pathogenic variants in the *PTPRC* (c.2204A > T, p.Asp735Val), *UNC119* (c.586T > A, p.Phe196Ile), and *IL21R* (c.481C > T, p.Arg161Cys) genes in the study patients (Table 3). Remarkably, all patients in our study carried the heterozygous genotype inherited from a parent who was a heterozygous genotype carrier but did not manifest the disease (Fig. 1). To date, much remains unknown in explaining how variants may influence phenotypic expression and how these variants exert their functional effects to cause disease. Monogenic diseases are thought to be caused by rare variants in genes that are directly associated with changes in protein function or dosage.^[29]^ However, the relationship between genotype and phenotype is complex; individuals with the same genotype can exhibit completely different clinical phenotypes,^[30–32]^ including the absence of clinical symptoms, even in members of the same family.^[33]^ Such variants can be identified as exhibiting incomplete penetrance, a phenomenon in which the genotype either causes the expected clinical phenotype or does not, or they may have variable expressivity, in which the same genotype can cause a variety of clinical symptoms. Genotype penetrance is determined by the proportion of individuals possessing a particular genotype who exhibit the clinical symptoms expected of that genotype.^[34,35]^ Incomplete penetrance and variable expressivity may explain the phenomenon that unaffected parents can transmit disease-causing variants to affected offspring.^[30]^ Gene expression is regulated by multiple overlapping mechanisms, which explains why an individual can carry many deleterious variants but remain unaffected.^[36]^ Humans also have other mechanisms to combat disease, including genetic modifications, which have been shown to help prevent humans from expressing the disease phenotype.^[37]^ Thus, many individuals carry pathogenic variants in highly penetrant monogenic disease genes but do not express the phenotype.^[38]^ Additionally, for most diseases, a combination of synergistic or antagonistic factors plays an important role in disease risk.^[39]^ Virolainen et al,^[40]^ pointed out that not all individuals carrying specific genetic variants may develop the corresponding disease.

Patient 1: Sanger sequencing results showed that a heterozygous variant (c.2204A > T, p.Asp735Val) in the *PTPRC* gene was identified in a healthy mother, sister, and patient (Fig. 1A). The *PTPRC* gene encodes the tyrosine phosphatase receptor protein type C (CD45), a transmembrane glycoprotein, present in most hematopoietic cells except mature red blood cells.^[41]^ CD45 contains an extracellular, a transmembrane, and 2 cytoplasmic catalytic domains (active domain 1 (D1) and catalytically impaired domain 2 (D2)).^[42]^ CD45 is an essential regulator of T and B cell antigen receptor signaling,^[43]^ which directly interacts with components of the antigen receptor or activates Src family kinases. CD45 controls immune responses by dephosphorylating several signaling molecules, including the Src family kinases (Lck and Fyn), the CD3z chain of the TCR, and the ZAP-70 kinase.^[42,43]^ An imbalance between the activities of protein tyrosine kinases and phosphatases can lead to immunodeficiency.^[43]^ CD45 controls the immune function by regulating lymphocyte survival, cytokine responses, and TCR signaling. Thus, altered CD45 may lead to severe combined immunodeficiency^[44]^ and T-B + SCID.^[28]^

The variant identified in patient P1 is located at position 735 in the β6 region of domain D1 of the PTPRC protein, which is the enzymatically active functional region.^[42]^ Analysis of the variant’s effect on the 3D structure of the PTPRC protein showed that replacing the amino acid Asp with Val resulted in the loss of a hydrogen bond between Asp at position 735 and Arg at position 738 (Fig. 1A). This substitution may have weakened the bonds between amino acids and affected the 3D structure of the protein, thereby affecting the function of CD45. Loss of CD45 function results in immunodeficiency that makes patients susceptible to infections, and elevated IgE may reflect a general immune dysfunction.

In patient 2, Sanger sequencing results showed that the heterozygous variant (c.586T > A, p.Phe196Ile) in the *UNC119* gene was inherited from the father, who also carried this variant in a heterozygous state but did not manifest the disease (Fig. 1B). Uncoordinated 119 (Unc119) is a Src (SH)3/SH2 homologous ligand that binds to CD3 and CD4, and functions to activate Lck and Fyn. Overexpression of Unc119 increases Lck/Fyn activity in T cells; in contrast, there is a significant reduction in Lck/Fyn activity, leading to decreased interleukin 2 production and cell proliferation in Unc119-deficient T cells.^[45]^ UNC119 is thought to have a functional role in all subsets of mature T cells and across different stages of differentiation.^[46]^ The first step in the TCR signaling is the activation of the receptor-associated kinases Src, Lck, and Fyn. However, the exact mechanism of this process remains unknown. A heterozygous missense mutation has been described in patients with idiopathic CD4 lymphocytopenia, an immunodeficiency disorder, that disrupts the Unc119-Lck interaction to stimulate Lck catalytic activity by the TCR.^[45]^ Patient have < 300 CD4 T cells/mL (20% of total T cells) and a history of recurrent sinusitis/otitis media, widespread onychomycosis, fungal dermatitis, oral lesions, and obstructive bronchitis with pneumonia. The patient’s cells are hyporesponsive to TCR stimulation, both localization and enzymatic activation of lymphocyte-specific kinase (Lck) impaired, leading to decreased cell proliferation.^[45]^

Decreased CD4 cell counts (941 cells × 10^6^/L, normal range 2330 to 3617 cells × 10^6^/L) and recurrent infections (pneumonia, otitis media, sepsis, dermatitis, and diarrhea) were also observed in patients in our study (Table 2). This result may be due to the amino acid change at position 196, a position in the conserved region between species (Fig. 1C), which changes the alpha-helical structure of domain 1 and affects the spatial structure of UNC119. This change leads to impaired activity of UNC119, which may lead to T cell inactivation and impaired immune response, making patients susceptible to infections, leading to increased IgE.

In patient 3, Sanger sequencing revealed that the patient carried a heterozygous variant (c.481C > T, p.Arg161Cys) in the IL21R gene inherited from her mother, who has the variant but did not manifest the disease (Fig. 1C). Effect analysis of the variant on the spatial structure of the IL21R protein showed that the substitution of Arginine by Cysteine resulted in the loss of 4 H-bonds between Arg161 and Pro171, Ser 170, and Val169, leading to a change in the spatial structure of IL21R protein (Fig. 1C). The patient’s test showed increased IgE but low IgG level; the patient had severe sepsis, dermatitis, and died (Table 2). Recent reports by Kotlarz et al^[47]^ and Salzer et al^[48]^ have implicated mutations in the IL21R gene as a novel cause of PID. Analysis of the tissue distribution of IL-21R demonstrated that it is predominantly expressed in lymphoid hematopoietic cells, such as T, B, NK cells, and myeloid cells.^[49]^ Expression of IL-21R on immune cells is tightly regulated depending on their activation state and environmental context. Furthermore, expression of IL-21R in nonhematopoietic cells has been reported in epithelial cells, keratinocytes,^[50]^ and fibroblasts.^[51]^ All IL-21R-deficient patients suffered from recurrent respiratory infections. Immunohistochemical testing revealed impaired B-cell proliferation and immunoglobulin class switching, reduced T-cell effector function, and natural killer cell dysfunction. Major clinical findings included recurrent pneumonia and otitis media; laboratory testing found increased IgE and decreased IgG levels.^[5,52]^

Understanding of novel variant genes takes responsibility for clinical outcomes in these patients, highlighting their effects on immune disorders, including recurrent infections and increased IgE levels, and emphasizing the need to diagnose accurately by molecular analysis to have appropriate interventions to enhance the effectiveness of treatment for patients and improve the quality of care for people with increased IgE, and related immune disorders.^[4,25]^

## 6. Conclusions

In this study, by WES, 3 novel pathogenic variants were detected in 3 PID patients with elevated IgE including c.2204A > T, p.Asp735Val in the *PTPRC* gene, c.586T > A, p.Phe196Ile in the *UNC119* gene, and c.481C > T, p.Arg161Cys in the *IL21R*. Our study has provided insights into novel variants in the genes associated with hyper-IgE in PID patients.

## Acknowledgments

We would like to thank the patients and their families who participated in this study.

## Author contributions

**Conceptualization:** Le Thi Minh Huong.

**Data curation:** Nguyen Thi Van Anh.

**Investigation:** Nguyen Thi Phuong Mai, Ngo Diem Ngoc, Nguyen Minh Thu, Nguyen Thi Thanh Ngan, Nguyen Thanh Hien.

**Methodology:** Nguyen Thi Kim Lien, Nguyen Van Tung.

**Software:** Nguyen Van Tung.

**Supervision:** Nguyen Huy Hoang.

**Visualization:** Le Thi Minh Huong.

**Writing – original draft:** Nguyen Thi Kim Lien.

**Writing – review & editing:** Nguyen Huy Hoang, Le Thi Minh Huong, Nguyen Thi Kim Lien, Tran Minh Dien.
